# Production of Hydrogels from Microwave-Assisted Hydrothermal Fractionation of Blackcurrant Pomace

**DOI:** 10.3390/gels9090674

**Published:** 2023-08-22

**Authors:** Natthamon Inthalaeng, Tom I. J. Dugmore, Avtar S. Matharu

**Affiliations:** Green Chemistry Centre of Excellence, Department of Chemistry, University of York, York YO10 5DD, UK; ni624@york.ac.uk (N.I.); tom.dugmore@york.ac.uk (T.I.J.D.)

**Keywords:** blackcurrant pomace, defibrillated cellulose, microwave-assisted hydrothermal treatment, hydrogels

## Abstract

The exploitation of unavoidable food supply chain wastes resulting from primary and secondary processing for chemicals, materials, and bioenergy is an important concept in the drive towards circular-based, resource-efficient biorefineries rather than petroleum refineries. The potential production of hydrogels (materials) from unavoidable food supply chain wastes, which are naturally rich in biopolymers such as cellulose, hemicellulose, pectin, and lignin, represents an interesting opportunity. However, these intertwined and interconnected biopolymers require separation and deconstruction prior to any useful application. Thus, this study aims to explore the formation of hydrogels from defibrillated celluloses (MW-DFCs) produced via acid-free stepwise microwave hydrothermal processing of blackcurrant pomace residues. Initially, pectin was removed from blackcurrant pomace residues (MW, 100–160 °C), and the resultant depectinated residues were reprocessed at 160 °C. The pectin yield increased from 2.36 wt.% (MW, 100 °C) to 3.07 wt.% (MW, 140 °C) and then decreased to 2.05 wt.% (MW, 160 °C). The isolated pectins were characterized by attenuated total reflectance infrared spectroscopy (ATR-IR), thermogravimetric analysis (TGA), and ^13^C NMR (D_2_O). The cellulosic-rich residues were reprocessed (MW, 160 °C) and further characterized by ATR-IR, TGA, and Klason lignin analysis. All the MW-DFCs contained significant lignin content, which prevented hydrogel formation. However, subsequent bleaching (H_2_O_2_/OH^−^) afforded off-white samples with improved gelling ability at the concentration of 5% *w*/*v*. Confocal laser microscopy (CLSM) revealed the removal of lignin and a more pronounced cellulosic-rich material. In conclusion, the microwave-assisted defibrillation of blackcurrant pomace, an exploitable unavoidable food supply chain waste, affords cellulosic-rich materials with the propensity to form hydrogels which may serve useful applications when put back into food products, pharmaceuticals, cosmetics, and home and personal care products.

## 1. Introduction

Blackcurrants (*Ribes nigrum*) are small purple berries that are rich in beneficial compounds such as ascorbic acid, flavonoids, and polyphenols, particularly anthocyanins and proanthocyanidins [1,2,3,4]. The fruits are used in juice production and, hence, produce significant amounts of waste as residual pomace, which still contains valuable compounds. For example, recent studies have focused on the extraction of anthocyanins from blackcurrant waste [2,4,5,6,7]. Blackcurrant pectin has been isolated, and it is different to citrus pectin as it is typically bound to anthocyanin and gives a broad range of biological properties [8]. Alba et al. report methods for fractionating dietary fibers, including soluble dietary fiber, insoluble dietary fiber, and lignin, from blackcurrants [9]. The extraction methods described in previous studies involve conventional heating, acidic conditions, alkaline conditions, and the use of chelating agents, which may have environmental implications [9].

Microwave hydrothermal treatment is known as a rapid and energy-efficient technique compared to the conventional methods. It allows for the production of biopolymers, e.g., pectin and cellulose, that are free from chemical or biological additives and have undergone defibrillation. By utilizing microwave energy, this process effectively and specifically eliminates hemicellulose, pectin, and amorphous cellulose from the biomass. This approach has been successfully applied to various biomass sources for production of pectineus and cellulosic materials [10,11,12,13,14]. 

Moreover, it has been found that cellulose recovered from MW extraction may lead to the formation of hydrogels. Several studies demonstrate that water and cellulose exhibit a strong affinity, allowing them to form gels [10,12,13,14,15]. The abundant hydroxyl groups present in cellulose play a vital role in creating a three-dimensional hydrogel by attracting and retaining water through hydrogen bonding [16]. These hydrogels derived from the biomass are in high demand for various applications, such as lubricants, contact lenses, hygiene products, and wound healing appendages [17].

**Figure 1:**
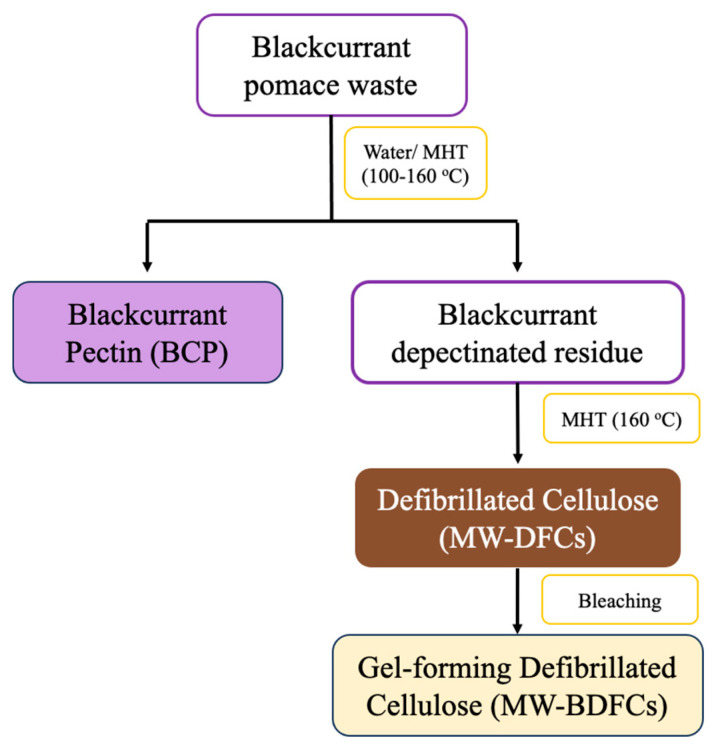
Schematic flow diagram for valorization of blackcurrant pomace.

Thus, given its large volume and chemical richness, blackcurrant pomace is an important economic biorefinery feedstock. Herein, we report the production of hydrogels from microwave-assisted hydrothermal fractionation of blackcurrant pomace (Figure 1). There is currently limited research available on pectin isolation and the defibrillation of cellulosic fibers from blackcurrant pomace via such a process. This process adds further value to the eco-friendly blackcurrant waste biorefinery via a zero waste approach. Initially, pectin is isolated using water (thermal heating) and microwave-assisted hydrothermal processing at temperatures ranging from 100 °C to 160 °C. Thereafter, the depectinated blackcurrant residues obtained are subjected to microwave hydrothermal reprocessing at 160 °C to yield defibrillated celluloses. The latter will be assessed for the ability to hold water (water-holding capacity) and the propensity to form hydrogels.

## 2. Results and Discussion

### 2.1. Pectin Yield and Characterization

#### 2.1.1. Pectin Yield and Degree of Esterification

**Figure 2:**
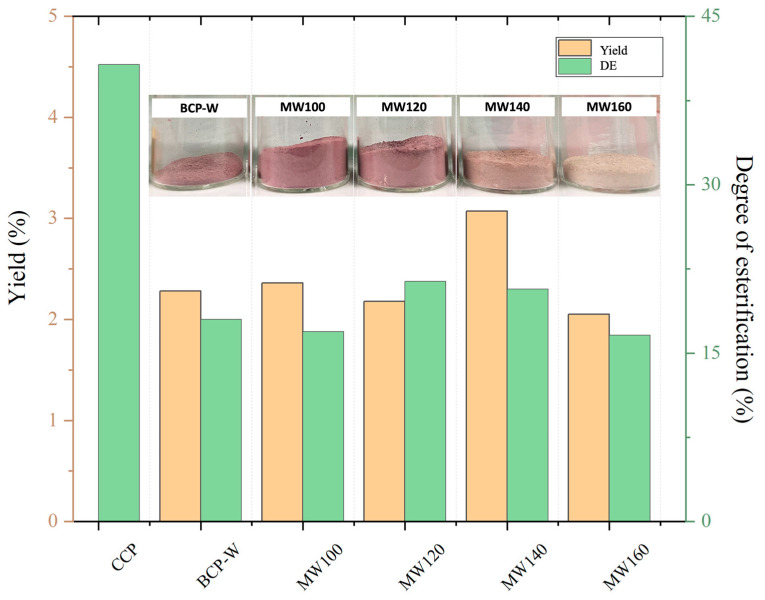
Summary of blackcurrant pectin (BCP) yield (wt.%) and degree of esterification (%) via various treatments. BCP-W: blackcurrant pectin obtained via water treatment; MW100-160: blackcurrant pectin obtained via microwave hydrothermal treatment at 100, 120, 140, and 160 °C; CCP: commercial citrus pectin.

The yield (wt.%) and degree of esterification (DE, %) of the isolated pectins from blackcurrant (BC) pomace under different conditions are presented in Figure 2. The highest pectin yield (3.07 wt.%) was obtained at a microwave temperature of 140 °C, which aligns well with the literature [18]. The DE, an essential characteristic of pectin, was calculated using the IR spectroscopic method, as reported by Demir et al. [19]. The DE of pectin increased with the increasing microwave temperature, peaking at 120 °C (DE, 21.3%), followed by a plateau at 140 °C; thereafter, it decreased at 160 °C (DE, 16.3%). Commercial citrus pectin (CCP) was used as a control or comparator sample. Based on the IR technique used, both the BC pomace pectin and the CCP were categorized as low methoxy (LM) pectin as their DE was less than 50%. This is consistent with the literature which reports BC pomace pectin with a DE of 33% [9]. Notably, the DE of BCP-W and all the microwave-derived pectins were significantly lower than that of CCP. Interestingly, all the derived pectins were pale pink in color, which is indicative of the presence of bound anthocyanins. However, as the microwave temperature increased to 160 °C, the pink color lessened, suggesting that higher temperatures may lead to the degradation of pectin-bound anthocyanins [20].

#### 2.1.2. Pectin Attenuated Total Reflection Infrared Spectroscopy (ATR-IR)

**Figure 3:**
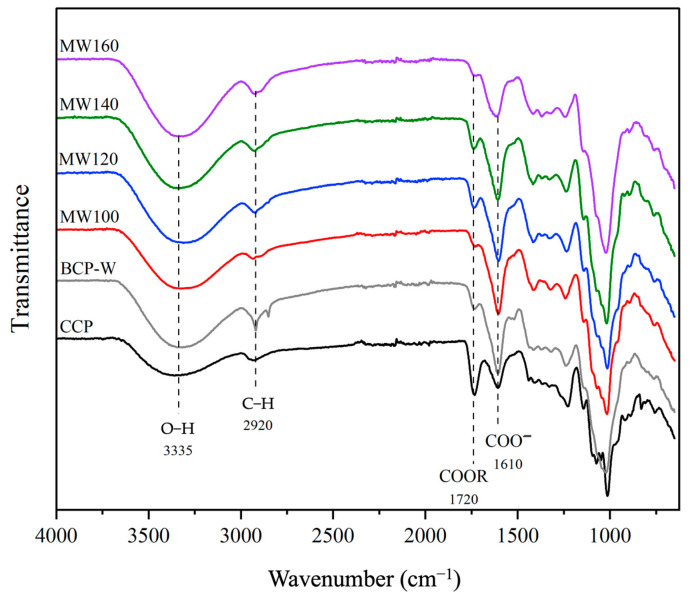
ATR−IR spectra of blackcurrant pectin (BCP) samples. CCP: commercial citrus pectin; BCP-W: blackcurrant pectin obtained via water treatment; and MW100−160: blackcurrant pectin obtained via microwave hydrothermal treatment at 100, 120,140, and 160 °C.

Figure 3 displays the ATR-IR spectra of BC pectin obtained from water extraction (BCP-W), microwave-assisted extraction (MW100-160), and commercial citrus pectin (CCP). The presence of pectin is evidenced by carbonyl absorption bands at 1720 cm^−1^ (C=OOCH_3_) and 1610 cm^−1^ (C=OOH), which were used to determine the DE. Furthermore, the spectra exhibited characteristic absorbance bands associated with the structure of pectin. For example, the broad O−H stretching band centered around 3335 cm^−1^ (indicating hydroxyl groups); the C−H stretching occurred at approximately 2920 cm^−1^, and there were intense C−O stretches.

#### 2.1.3. Pectin Thermogravimetric Analysis (TGA)

**Figure 4:**
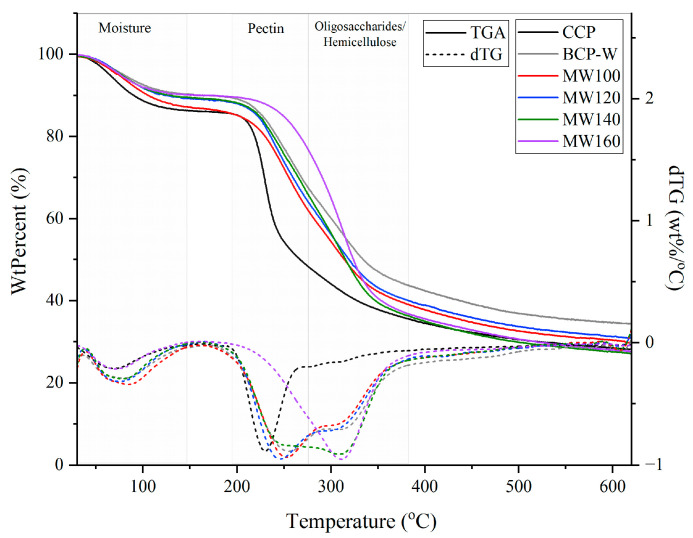
TGA and dTG curves of blackcurrant pectin (BCP) samples. CCP: commercial citrus pectin; BCP-W: blackcurrant pectin obtained via water treatment; and MW100-160: blackcurrant pectin obtained via microwave hydrothermal treatment at 100, 120,140, and 160 °C.

TGA was performed to assess the thermal stability of the derived blackcurrant pectins in comparison with the commercial citrus pectin (CCP) (Figure 4). All the BC derived-pectin samples displayed three distinct degradation temperatures: 25–150 °C, 220–280 °C, and 280–350 °C, corresponding to loss of moisture and volatile compounds, pectin, and hemicellulose, respectively. However, as CCP has been industrially purified it demonstrated a less-pronounced mass loss between 280 and 350 °C, whereas BC pectin exhibited a higher mass percentage loss within this temperature range. Interestingly, the derivative thermogravimetric analysis (dTG) of microwave (MW) pectin displayed a shift in the decomposition peak from 230–310 °C to 250–350 °C with increasing temperature. The most pronounced shift occurred for pectin processed at 160 °C, suggesting a significant change in the structure of pectin or hydrolysis [18].

#### 2.1.4. Pectin Carbon Nuclear Magnetic Resonance Spectroscopy (^13^C NMR)

**Figure 5:**
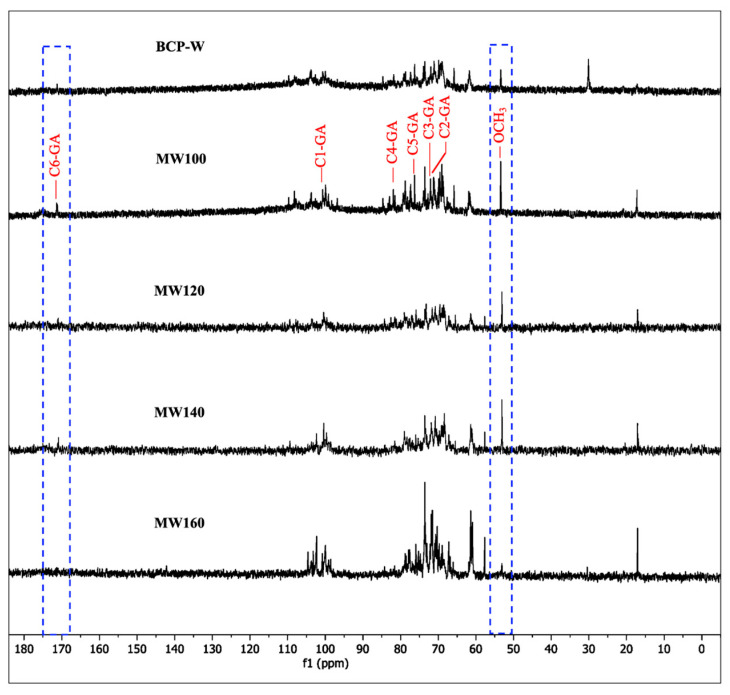
^13^C NMR spectra (in D_2_O) of blackcurrant pectin (BCP) samples. BCP-W: blackcurrant pectin obtained via water treatment; MW100-160: blackcurrant pectin obtained via microwave hydrothermal treatment at 100, 120, 140, and 160 °C.

Solution ^13^C NMR spectroscopic studies (in D_2_O) were performed on the pectins derived from the BCP-W and MW extractions; the results are shown in Figure 5. The resonances in the regions of 60–80 ppm and 97–105 ppm were characteristic of C2–C5 of monosaccharides and C1 (anomeric) of monosaccharides, respectively. Additionally, the resonances at 53.1 and 170.8 ppm were attributed to the CH_3_ of methyl ester and C-6 of galacturonic acid, respectively [8,18,21,22]. Notably, these resonances decreased when the MW processing temperature exceeded 140 °C, which is indicative of thermal degradation and the decarboxylation of homogalacturonan (HG).

### 2.2. Hydrogels Formation and Defibrillated Cellulose Characterization

#### 2.2.1. Hydrogel Formation of Defibrillated Cellulose

**Figure 6:**
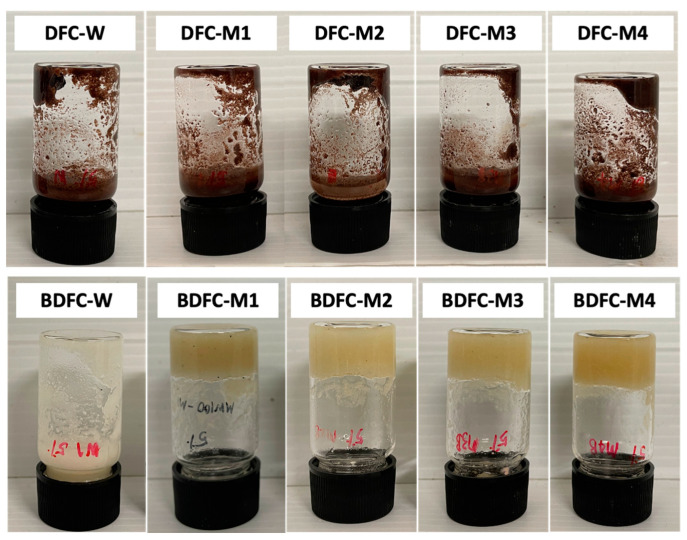
Hydrogel formation of defibrillated cellulose samples. DFC: defibrillated cellulose (**top**); BDFC: bleached defibrillated cellulose (**bottom**); W: sample obtained from water pre-treatment; and M1–M4: samples obtained from microwave hydrothermal pre-treatment from 100 to 160 °C, respectively.

The propensities of unbleached defibrillated celluloses (DFCs) and bleached defibrillated celluloses (BDFCs) to form hydrogels at 5% *w*/*v* in water are highlighted in Figure 6. Notably, all the DFC samples and the BDFC-W failed to form hydrogels of any note. However, all the microwave-processed BDFCs produced stable hydrogels that remained unchanged for approximately 30 min, opening possible opportunities in healthcare, coatings, etc. [23]. With these interesting preliminary results, the DFCs were characterized for their cellulose, hemicellulose, and Klason lignin content as well as their ability to hold water (water-holding capacity) in order to ascertain any links to hydrogel formation.

#### 2.2.2. Klason Lignin and Water-Holding Capacity (WHC)

**Figure 7:**
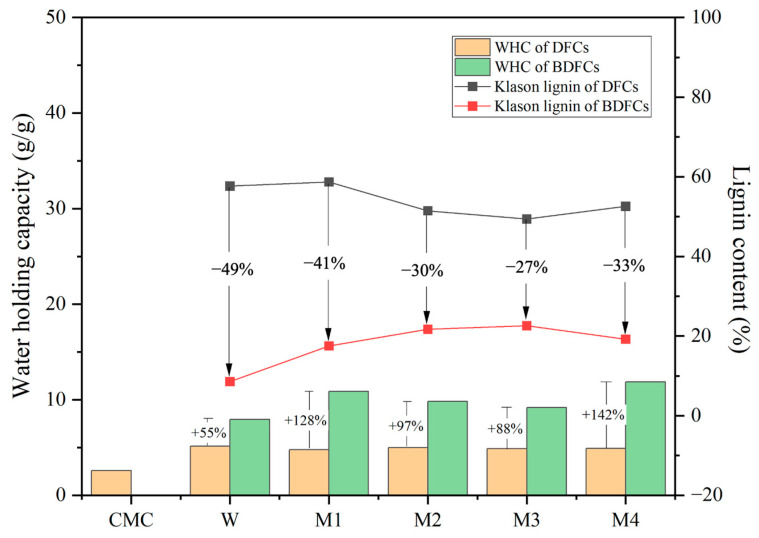
Klason lignin analysis and water-holding capacity (WHC) of defibrillated cellulose samples. CMC: commercial microcrystalline cellulose; DFC: defibrillated cellulose; BDFC: bleached defibrillated cellulose; W: sample obtained from water pre-treatment; and M1–M4: samples obtained from microwave hydrothermal pre-treatment from 100 to 160 °C, respectively.

The Klason lignin content and water-holding capacity (WHC) of the DFC and BDFC samples are summarized in Figure 7. The Klason lignin content of the DFC samples fluctuated around 50 wt.% and decreased by 27–49% after bleaching. As lignin is hydrophobic in nature, the significant Klason lignin content of approximately 50 wt.% may be linked to the lower ability to hold water. The WHC of commercial microcrystalline cellulose (CMC) was investigated as a control and found to be 2.6 g H_2_O/g sample. The WHC of the DFCs was twice that of CMC and remained relatively constant (approximately 4.9 g H_2_O/g sample), regardless of the pre-treatment method used. These values, while higher than for CMC, are lower when compared to previous studies on cellulosic materials derived from lignocellulosic or pectin-rich biomass [10,12]. Interestingly, the BDFC samples demonstrated a doubling in their WHC compared with their unbleached counterparts, i.e., DFC, approx. 4.9 g H_2_O/g sample versus BDFC, approx. 9.9 g H_2_O/g sample. Compared with similar defibrillated unbleached samples in the literature, bleaching has a positive influence on WHC [14,24].

#### 2.2.3. Attenuated Total Reflection Infrared Spectroscopy (ATR-IR) of Defibrillated Celluloses

**Figure 8:**
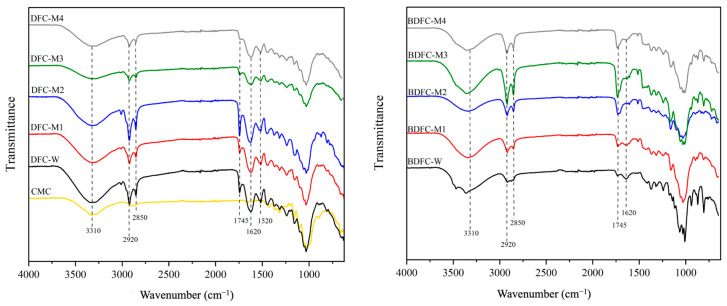
ATR-IR spectra of defibrillated cellulose samples. CMC: commercial microcrystalline cellulose; DFC: defibrillated cellulose (left); BDFC: bleached defibrillated cellulose (right); W: sample obtained from water pre-treatment; and M1–M4: samples obtained from microwave hydrothermal pre-treatment from 100 to 160 °C, respectively.

The ATR-IR spectra of the isolated DFC and BDFC samples from the depectinated blackcurrant pomace compared with those of the CMC are displayed in Figure 8. All the MFC samples showed the main characteristic absorption bands associated with the structure of cellulose: broad O-H stretching centered at around 3330 cm^−1^ (hydroxyl groups), C-H stretching at approximately 2920 cm^−1^, C-O stretching vibrations at roughly 1050 cm^−1^, and C-H out-of-plane vibration at 898 cm^−1^ [25]. In addition, the evidence for the presence of hemicellulose, pectin, and lignin were observed in all the isolated DFCs. A relatively small absorbance band at around 1520 cm^−1^ may be attributed to the C-H bending vibration of lignin [26], which was notably absent in the BDFC samples, suggesting that the lignin had been entirely removed. The absorbance bands at around 1735 cm^−1^ and 1620–1640 cm^−1^ were assigned to the carbonyl group in hemicellulose and pectin but, at times, may have been confused with residual water [27].

#### 2.2.4. Thermogravimetric Analysis (TGA) of Defibrillated Cellulose Samples

**Figure 9:**
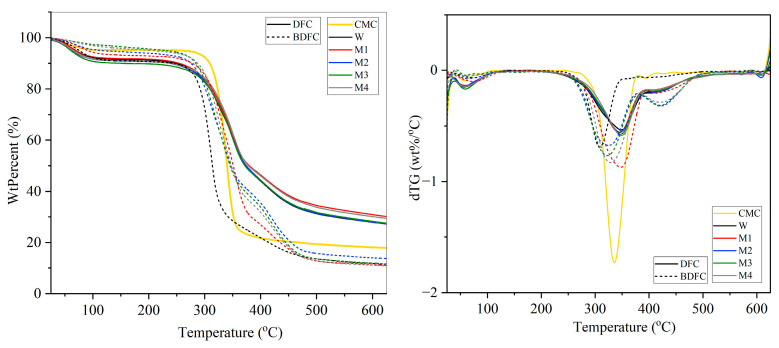
TGA (**left**) and dTG (**right**) curves of defibrillated cellulose samples. CMC: commercial microcrystalline cellulose; DFC: defibrillated cellulose; BDFC: bleached defibrillated cellulose; W: sample obtained from water pre-treatment; and M1–M4: samples obtained from microwave hydrothermal pre-treatment from 100 to 160 °C, respectively.

The thermal stability of the defibrillated celluloses from blackcurrant pomace residue was determined using TGA as shown in Figure 9. The degradation temperatures for the moisture and volatile compounds, hemicellulose, cellulose, and lignin ranged between 25 and 150 °C, 220 and 315 °C, 315 and 400 °C, and 160 and 600 °C, respectively. An initial mass loss from 25 °C to 120 °C was due to the evaporation of moisture. The rapid mass loss at 300 °C to 380 °C was due to the thermal degradation of cellulose. The third region of mass loss occurred after 380 °C which may attributed to the degradation of aromatic structures and organic polymers such as lignin [28,29,30,31]. The thermal degradation of DFCs displayed similar patterns, suggesting that the initial pre-treatment conditions did not have any impact on cellulose structure or its thermal stability. The microwave hydrothermal reprocessing at 160 °C gave a sample rich in cellulosic matter, but it still had residual lignin. These outcomes are consistent with the existing literature, which indicates that the isolated celluloses, when subjected to MHT (microwave hydrothermal treatment) up to 216 °C, still showed the presence of significant amounts of lignin [10]. Lignin is more resistant to thermal degradation than carbohydrate biopolymers, and its decomposition temperature spans a wide range, reaching up to 900 °C [32]. Consequently, the presence of lignin in the DFC samples has a discernible impact on their degradation temperature (T_d_), with a 10 °C increase compared to standard CMC. In addition, all the BDFC samples, obtained after bleaching, exhibited a T_d_ similar to that of CMC. The final residues observed after thermal degradation at 625 °C indicated that the DFC samples had approximately 20% higher final residues compared to the BDFC samples. A lower content of final residues suggests a higher purity of cellulose [33,34]. This observation is consistent with the findings of the Klason lignin content and the ATR-IR analysis reported earlier, indicating that the BDFC samples have higher cellulose content and lower lignin content compared to the DFC samples.

#### 2.2.5. Confocal Laser Scanning Microscopy (CLSM) Analysis

**Figure 10:**
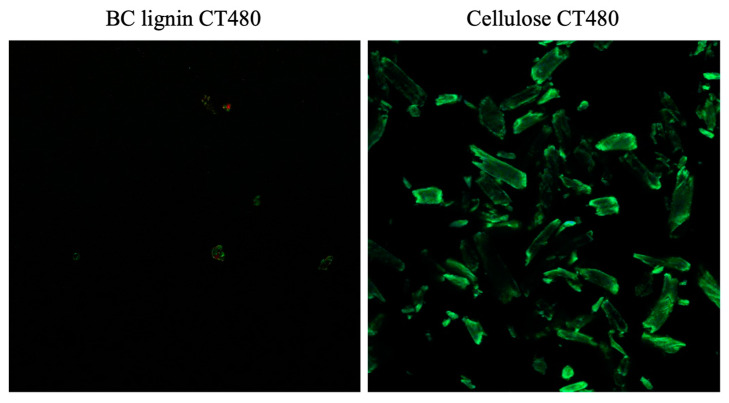
Confocal laser scanning microscopy (CLSM) images of extracted BC lignin stained with CT480 (**left**) and commercial cellulose stained with CT480 (**right**).

Defibrillated blackcurrant samples stained with Carbotrace 480 (CT480) were visually examined via CLSM using the method described by Zitzmann et al. [15]. As controls, the isolated lignin from the blackcurrant pomace and CMC were also stained with CT480. The results obtained from the CLSM showed that CMC stained with CT480 emitted a vivid green color, while CT480 staining on extracted blackcurrant lignin yielded almost no emission (Figure 10). This observation suggests that CT480 exhibits a strong affinity for cellulose, which can be explained by the interaction facilitated by Carbotrace, a class of fluorescence oligothiophene molecules. Oligothiophenes exhibited specific binding tendencies to molecules consisting of glycosidic linkages. The binding of cellulose and oligothiophene induced the flattening of the thiophene moiety, resulting in increased fluorescent emission [35,36]. In contrast, Carbotrace with lignin exhibited almost no fluorescent emission due to the aromatic moieties in lignin strongly interacting with the thiophene moiety and affecting the electron quenching of the oligothiophene fluorescent molecules, resulting in a decrease in fluorescent emission [37].

**Figure 11:**
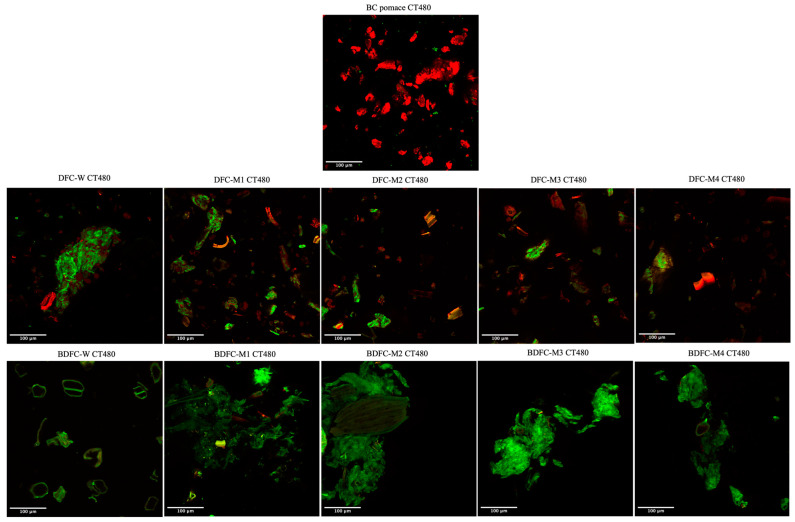
Confocal laser scanning microscopy (CLSM) images of BC pomace, DFC samples, and BDFC samples stained with CT480.

When the DFC and BDFC samples, as well as the raw blackcurrant pomace residue, were stained with CT480, a bright green emission from the interaction with cellulose was detected, whilst a red color was applied to visualize the residue in the samples (Figure 11). The significant red color observed in the BC pomace indicated lower cellulose content compared to the BC samples obtained after MHT. However, in the DFC samples, there was no discernible trend in cellulose content with increasing MW temperature pre-treatment, suggesting that MW reprocessing at 160 °C can effectively purify depectinated BC residues to a similar level. Furthermore, the bleaching process played a crucial role in purifying cellulose, as evidenced by a more intense green emission and reduced red emission post-bleaching. The presence of more cellulose aggregates were observed in the MW-BCFC samples, whereas the BDFC-W showed independent cellulose entities. Thus, the BDFC samples with higher purity and more aggregated cellulose structures had a greater propensity to form hydrogels.

## 3. Conclusions

Our study demonstrates that blackcurrant pomace residues can be fractionated to yield pectin and defibrillated cellulose using an acid-free microwave hydrothermal treatment. The produced blackcurrant pectin exhibited a low degree of esterification (16.6–21.4%). The defibrillated celluloses that were devoid of pectin demonstrated the capacity to hold water but lacked the ability to form a hydrogel. Through further bleaching of the defibrillated celluloses using H_2_O_2_ under alkaline conditions, their water-holding capacity was significantly enhanced. The bleached samples can hold water at a rate of 9.9 g H_2_O/g sample, which is twice the capacity of the unbleached samples (4.9 g H_2_O/g sample), and four times higher than the commercial microcrystalline cellulose (2.6 g H_2_O/g sample). Additionally, the bleached samples display stable hydrogel formation at a concentration of 5% *w*/*v*. These materials hold promise for applications in pharmaceuticals, packaging, food, cosmetics, and home and personal care. In conclusion, this work not only isolates pectin and cellulosic materials but also introduces a novel and eco-friendly approach towards achieving a zero waste blackcurrant biorefinery.

## 4. Materials and Methods

### 4.1. Materials

Blackcurrant (BC) pomace residue was industrially sourced from Lucozade Ribena Suntory, Coleford, which is a part of Suntory Beverage & Food Great Britain and Ireland. The as-received pomace was air-dried at ambient temperature for one week and then milled using a coffee grinder into granular particles (≤2 mm). 

### 4.2. Preparation of Depectinated BC Residues

The different pre-treatment conditions (water and microwave hydrothermal pre-treatment) were used for the removal of pectin and some non-cellulosic contents from BC pomace residues.

#### 4.2.1. Water Pre-Treatment

Milled BC pomace residue (40 g) was mixed with deionized water (200 mL) and heated at a reflux temperature for 2 h.

#### 4.2.2. Microwave Pre-Treatment

Milled BC pomace residue (20 g) was treated with deionized water (300 mL) and processed under the microwave conditions (Milestone Synthwave Microwave, 1500 W, 2.45 GHz) at 100 °C, 120 °C, 140 °C, or 160 °C for 30 min (15 min ramping time and 15 min holding time).

The cooled mixtures were filtered through a stainless steel mesh under a vacuum. The liquid fraction was retained for pectin precipitation, whilst the depectinated residues were washed with boiling water, hot ethanol, ethanol, and acetone and air-dried for 2 days.

### 4.3. Isolation of Defibrillated Cellulose

The appropriate depectinated BC pomace residue (20 g) and deionized water (300 mL) were added into a PTFE microwave vessel and subjected to microwave irradiation (160 °C, 15 min ramping time and 15 min holding time). Thereafter, the cooled mixture was filtered (Buchner), and the residues were washed with boiling water, hot ethanol, ethanol, and acetone. The washed residues were air-dried for 2 days, manually ground, sieved (250 µm mesh stainless filter), and defined as unbleached defibrillated cellulose samples (MW-DFCs). 

The MW-DFCs were further bleached with a slightly modified method suggested by Alba et al. [9,38]. The MW-DFCs were bleached with 6% *v*/*v* H_2_O_2_ with the solid-to-liquid ratio of 1:20 (g/mL) at pH 11.5 (adjusted by 6M NaOH), 60 °C for 2 h, followed by room temperature for 16 h. The bleached MW-DFCs (MW-BDFCs) were centrifuged at 3500 rpm for 20 min. The solid fractions were washed with water (3 times), followed by 5% *w*/*v* acetic acid, water, ethanol, and acetone, and air-dried for 2 days, then manually ground and sieved (250 µm mesh stainless filter).

### 4.4. Characterization of BC Pectin, MW-DFCs, and MW-BDFCs

#### 4.4.1. Carbon Nuclear Magnetic Resonance (^13^C NMR) Analysis

^13^C NMR were recorded and operated at 125 MHz, 343 K, with 23,000 scans on a Bruker AVIIIHD600 spectrometer. The pectin samples (75 mg) were dissolved in deuterium oxide (1.5 mL, 50 mg/mL) and allowed to be mixed at 40 °C overnight. The data were processed by MestReNova x64 software version 14.3.1.31739. 

#### 4.4.2. Attenuated Total Reflection Infrared (ATR-IR) Spectroscopy Analysis

ATR-IR was carried out using a Perkin Elmer Spectrum 400 IR. The spectra were scanned from 4000 to 600 cm^−1^. Before recording the data, the sapphire window and the lid were cleaned with isopropanol. The spectra for moisture and carbon dioxide correction were derived from the surrounding atmosphere. 

#### 4.4.3. Thermogravimetric Analysis (TGA)

TGA analysis was performed using the Stanton Redcroft STA625 under a flow of nitrogen gas. About 10 mg of pectin or DFC samples was placed into the aluminum pan and measured against the empty reference aluminum pan under the increasing temperature from room temperature to 625 °C at the rate of 10 °C/min. The data were processed using Origin software version 2022bSr1.

#### 4.4.4. Klason Lignin Analysis

Klason lignin was determined by the NREL method with a slight modification [39]. The DFC sample (100 mg, W) was hydrolyzed with 72% H_2_SO_4_ (1 mL) in a shaking water bath (40 °C, 2 h). Afterwards, 28 mL of water was added to reduce the acid concentration to 4% H_2_SO_4_; then, it was autoclaved at 121 °C for 1 h. The hydrolyzed sample was filtered through a filter crucible (W_0_), washed with distilled water, and then put in an oven to dry at 105 °C overnight. The crucible with solid residue was cooled down in a desiccator, and the weight was recorded (W_1_). The water-holding capacity was calculated using the following Equation (1):

(1) $$Insoluble\;lignin\left(\%\right)=\frac{W_{1}-W_{0}}{W}\times 100$$

#### 4.4.5. Water-Holding Capacity (WHC)

WHC was determined using the methods of Romruen et al. and Zain et al. [34,40], with a slight modification. The DFC samples (0.2 g, W_0_) and deionized water (10 mL) were placed into a centrifuge tube (W_T_) and vortexed (2000 rpm, 1 min), followed by sonication (30 °C, 20 min). The mixture was kept at room temperature overnight and was then centrifuged (3900 rpm, 20 min). The supernatant was carefully discarded while the wet sediment and tube were weighed (W_S_). The weight of the water held in the samples was W_1_ (W_1_ = W_S_ − W_T_). The water-holding capacity was calculated using the following Equation (2):

(2) $$Water\;holding\;capacity=\frac{W_{1}-W_{0}}{W_{0}}$$

#### 4.4.6. Confocal Laser Scanning Microscopy (CLSM) Analysis

CLSM analysis of defibrillated cellulose samples using Carbotrace 480 was processed using the protocol of Zitzmann et al. [15]. The samples (20 mg) were mixed and incubated with 50 µL of Carbotrace 480: phosphase buffer solution pH 7.4 (ratio 1:1000) for 30 min. The CLSM images were acquired using a Zeiss LSM980 confocal microscope, AxioObserver Z1, using ZEN 3.4 (blue edition) software and either an EC Plan-Neofluar 10×/0.3 or a Plan Apochromat 20×/0.8 objective. All the samples were excited with a 405 nm laser using a 405 nm main beam splitter, and the emissions were collected from 411 to 694 nm in bins of 8.9 nm. 

#### 4.4.7. Hydrogel Formation

DFC hydrogels were formed by suspending DFC samples with deionized water (2.5 mL) at a concentration of 5% *w*/*v* and mixing via vortexing (2000 rpm, 1 min) and sonication (30 °C, 20 min). The mixtures were allowed to stand for 18 h at room temperature. The stability of the hydrogel was evaluated by inverting the gel vial for 30 min to qualitatively assess the gel strength.

### 4.5. Statistical Analysis

The data were analyzed for significant differences (*p*-value < 0.05 was considered significant), with a confidence interval of 95%, using analysis of variance (ANOVA) and Fisher’s least significant difference (LSD) test. The analysis was performed using IBM SPSS Statistics software version 28.0.1.1. All the analyses were conducted in duplicate. The detailed breakdown of the statistical analysis is given in the ESI; Tables S1.1, S1.2, S2.1, S2.2, S3.1 and S3.3.

The examination of the pectin yield through analysis of variance (ANOVA) revealed a significant variability in yield across different pectin extraction conditions (with a *p*-value < 0.05, n = 3). Hence, the selection of extraction parameters, which notably encompassed conventional water extraction and MHT varying from 100 to 160 °C, exerted a discernible influence on the resultant pectin yield. To discern disparities in pectin yield among the various sample pairs, the least significant difference (LSD) test was employed. The pectin yields exhibited a range of 2.04% to 3.07%. The results summarized in Table S1.2 (ESI) indicated that the MW140 pectin yield was significantly higher than that of the other samples at the *p*-value level of 0.05, while the pectin produced via conventional water extraction and MHT at 100, 120, and 160 °C yielded no significant difference in pectin yield.

The ANOVA conducted on the WHC of the cellulose samples indicated a variation in WHC between each sample (with a *p*-value < 0.05, CMC and DFCs; n = 3, BDFCs; n = 2). The LSD test was employed to distinguish the mean difference between each sample. The water-holding capacity (WHC) values exhibited a notable variation among the different cellulose samples. The average WHC values for the commercial microcrystalline cellulose (CMC), DFCs, and BDFCs were 2.6, 4.77–5.14, and 7.95–11.53 g H_2_O/g, respectively (see Table S2.2, ESI). Distinct differences were evident between the CMC and both the DFCs and BDFCs. Within the DFC group, the WHC did not show significant divergence, whereas for the BDFC group, significant variation in WHC was observed. The mean difference in WHC between the CMC and either the DFCs or BDFCs ranged from 2.17 to 2.53 g H_2_O/g and from 5.35 to 8.93 g H_2_O/g, respectively. Notably, the WHC of the DFCs was approximately double that of the CMC, while the WHC of the BDFCs was notably higher, ranging from 3.5 to 4.4 times that of the CMC. Furthermore, when comparing the samples subjected to the same extraction conditions, the WHC of the bleached samples (BDFCs) demonstrated an approximately 1.5–2.3-fold increase compared to the unbleached samples (DFCs).

The Klason lignin content of the celluloses was analyzed in duplicate (n = 2). The resultant ANOVA revealed a significant difference in the Klason lignin content in the cellulose with the *p*-value < 0.05, n = 2 (Table S3.1, ESI). Thus, the LSD test was performed to distinguish the differences in the Klason lignin content between each of the cellulose samples (Table S3.2, ESI). Significant variations were observed in the Klason lignin content between the DFCs and BDFCs, encompassing a range of 49.38% to 58.27% and 8.58% to 22.59%, respectively. Following the bleaching process, the Klason lignin content of the bleached samples (BDFCs) underwent a substantial reduction of 27% to 49%. This decrease in Klason lignin content within the cellulose samples indicated an enhancement in water-holding capacity (WHC). While the lignin content exhibited an influence on both the WHC of the cellulose samples and, subsequently, the hydrogel formation, it is noteworthy that the BDFC-W sample, characterized by the lowest lignin content (8.58%), was unable to facilitate hydrogel formation. Therefore, a more comprehensive assessment, encompassing cellulose characterization and additional analyses, is imperative for further elucidation.

## Data Availability

The data presented in this study are available within the article.

## Supplementary Materials

The following supporting information can be downloaded at: https://www.mdpi.com/article/10.3390/gels9090674/s1, Table S1.1: Analysis of variance (ANOVA) of pectin yield via different extraction conditions; Table S1.2: Summary of the mean difference of yield between pairs of samples using the LSD test; Table S2.1: Analysis of variance (ANOVA) of WHC of cellulose; Table S2.2: Summary of the mean difference in WHC of cellulose using the LSD test; Table S3.1: Analysis of variance (ANOVA) of Klason lignin content in cellulose samples; Table S3.2: Summary of mean difference of Klason lignin content in each cellulose samples using LSD test.
