# Supplementary material for: Production of Hydrogels from Microwave-Assisted Hydrothermal Fractionation of Blackcurrant Pomace
Source: Gels. 2023 Aug 22;9(9):674. doi: 10.3390/gels9090674 (PMC10530458; doi:10.3390/gels9090674)
Supplement: Supplementary file 1 [file gels-09-00674-s001.zip › gels-2563977-supplementary.pdf]

1. Statistical analysis of Pectin yield

Table S1.1 Analysis of variance (ANOVA) of pectin yield via different extraction conditions

ANOVA

Pectin Yield

	Sum of Squares	df	Mean Square	F	P value
Between Groups	1.919	4	.480	4.434	.026
Within Groups	1.082	10	.108		
Total	3.001	14			

The examination of pectin yield through analysis of variance (ANOVA) revealed significant variability in yield across different pectin extraction conditions (with a *P-value* < 0.05, n = 3). Hence, the selection of extraction parameters, notably encompassing conventional water extraction and MHT varying from 100 to 160°C, exerted a discernible influence on the resultant pectin yield. To discern disparities in pectin yield among various sample pairs, the least significant difference (LSD) test was employed.

Table S1.2 Summary of the mean difference of yield between pairs of samples using the LSD test.

Sample	Mean (%)	BCP-W	MW100	MW120	MW140	MW160
		2.28	2.33	2.18	3.07	2.04
BCP-W	2.28	-	0.05	0.10	0.79*	0.23
MW100	2.33		-	0.15	0.74*	0.28
MW120	2.18			-	0.89*	0.13
MW140	3.07				-	1.02*
MW160	2.04					-

*The mean difference is significant at the *p-value* level of 0.05

The pectin yields exhibited a range of 2.04% to 3.07%. The results summarized in **Table S1.2** indicated that the MW140 pectin yield was significantly higher than other samples at the p-value level of 0.05, while the pectin produced via conventional water extraction, MHT at 100, 120 and 160 ^{o}C yielded no significant difference in pectin yield.

2. Statistical analysis of Water holding capacity (WHC) of cellulose

Table S2.1 Analysis of variance (ANOVA) of WHC of cellulose

ANOVA

WHC of cellulose

	Sum of Squares	df	Mean Square	F	P value
Between Groups	211.979	10	21.198	349.488	<.001
Within Groups	1.031	17	.061		
Total	213.010	27			

The ANOVA was conducted on WHC of cellulose. The results indicated a variation in WHC between each sample (with a *P-value* < 0.05, CMC and DFCs; n=3, BDFCs; n=2). The LSD test was employed to distinguish the mean difference between each sample.

Table S2.2 Summary of the mean difference in WHC of cellulose using the LSD test.

Sample	Mean (g/g H_2O)	CMC	DFC-W	DFC-M1	DFC-M2	DFC-M3	DFC-M4	BDFC-W	BDFC-M1	BDFC-M2	BDFC-M3	BDFC-M4
		2.60	5.14	4.77	5.00	4.89	4.91	7.95	10.88	9.83	9.17	11.53
CMC	2.60	-	2.53*	2.17*	2.39*	2.29*	2.30*	5.35*	7.22*	8.28*	6.56*	8.93*
DFC-W	5.14		-	0.37	0.14	0.25	0.23	2.82*	5.75*	4.70*	4.03*	6.40*
DFC-M1	4.77			-	0.23	0.12	0.14	3.19*	6.12*	5.06*	4.40*	6.76*
DFC-M2	5.00				-	0.11	0.09	2.96*	5.89*	4.83*	4.17*	6.53*
DFC-M3	4.89					-	0.02	3.06*	5.99*	4.94*	4.28*	6.64*
DFC-M4	4.91						-	3.05*	5.98*	4.93*	4.26*	6.63*
BDFC-W	7.95							-	2.93*	1.88*	1.21*	3.58*
BDFC-M1	10.88								-	1.05*	1.72*	0.65*
BDFC-M2	9.83									-	0.66*	1.70*
BDFC-M3	9.17										-	2.37*
BDFC-M4	11.53											-

*The mean difference is significant at the *p-value* of 0.05

The water holding capacity (WHC) values exhibited notable variation among different cellulose samples. The average WHC values for commercial microcrystalline cellulose (CMC), DFCs, and BDFCs were 2.6, 4.77 – 5.14, and 7.95 – 11.53 g H_2O/g, respectively (see **Table S2.2**). In terms of WHC, distinct differences were evident between CMC and both DFCs and BDFCs. Within the DFC group, the WHC did not show significant divergence, whereas for the BDFC group, significant variation in WHC was observed. The mean difference in WHC between CMC and either DFCs or BDFCs ranged from 2.17 to 2.53 g H_2O/g and from 5.35 to 8.93 g H_2O/g, respectively. Notably, the WHC of DFCs was approximately double that of CMC, while the WHC of BDFCs was notably higher, ranging from 3.5 to 4.4 times that of CMC. Furthermore, when comparing samples subjected to the same extraction conditions, the WHC of bleached samples (BDFCs) demonstrated an approximate 1.5–2.3-fold increase compared to unbleached samples (DFCs).

3. Statistical analysis of Klason lignin content in cellulose samples

Table S3.1 Analysis of variance (ANOVA) of Klason lignin content in cellulose samples

ANOVA

Lignin content

	Sum of Squares	Df	Mean Square	F	Sig.
Between Groups	6833.907	9	759.323	637.315	<.001
Within Groups	11.914	10	1.191		
Total	6845.821	19			

The Klason lignin content of celluloses were analyzed in duplicate (n=2). The resultant ANOVA revealed significant different in Klason lignin content in cellulose with the *p-value* < *0.05*, n = 2 (**Table S3.1**). Thus, the LSD test was performed to distinguish the mean difference of Klason lignin content in each cellulose samples (**Table S3.2**).

Table S3.2 Summary of mean difference of Klason lignin content in each cellulose samples using LSD test.

Sample	Mean (%)	DFC-W	DFC-M1	DFC-M2	DFC-M3	DFC-M4	BDFC-W	BDFC-M1	BDFC-M2	BDFC-M3	BDFC-M4
		57.66	58.27	51.44	49.38	52.57	8.58	17.52	21.68	22.59	19.22
DFC-W	57.66	-	0.61	6.22*	8.29*	5.10*	49.09*	40.15*	35.98*	35.07*	38.44*
DFC-M1	58.27		-	6.83*	8.90*	5.71*	49.70*	40.76*	36.59*	35.68*	39.05*
DFC-M2	51.44			-	2.07	1.13	42.87*	33.93*	29.76*	28.85*	32.22*
DFC-M3	49.38				-	3.19*	40.80*	31.86*	27.70*	26.78*	30.16*
DFC-M4	52.57					-	43.99*	35.05*	30.89*	29.98*	33.35*
BDFC-W	8.58						-	8.94*	13.11*	14.02*	10.65*
BDFC-M1	17.52							-	4.16*	5.08*	1.71
BDFC-M2	21.68								-	0.91	2.46*
BDFC-M3	22.59									-	3.37*
BDFC-M4	19.22										-

*The mean difference is significant at the *p-value* of 0.05

Significant variations were observed in the Klason lignin content between DFCs and BDFCs, encompassing a range of 49.38% to 58.27% and 8.58% to 22.59%, respectively. Following the bleaching process, the Klason lignin content of the bleached samples (BDFCs) underwent a substantial reduction of 27% to 49%. This decrease in Klason lignin content within the cellulose samples indicated an enhancement in water holding capacity (WHC). While the lignin content exhibited an influence on both the WHC of cellulose samples and subsequently on hydrogel formation, it's noteworthy that the BDFC-W sample, characterized by the lowest lignin content (8.58%), was unable to facilitate hydrogel formation. Therefore, a more comprehensive assessment, encompassing cellulose characterization and additional analyses, is imperative for further elucidation.
